# Supplementary material for: Half a century of rising extinction risk of coral reef sharks and rays
Source: Nat Commun. 2023 Jan 17;14:15. doi: 10.1038/s41467-022-35091-x (PMC9845228; doi:10.1038/s41467-022-35091-x)
Supplement: Supplementary file 1 — Supplementary Information [file 41467_2022_35091_MOESM1_ESM.pdf]

Supplementary Information

Half a century of rising extinction risk of coral reef sharks and rays

Author list: C. Samantha Sherman*[1,2], Colin A. Simpfendorfer*[3], Nathan Pacoureau, Jay H. Matsushiba[1], Helen F. Yan, Rachel H. L. Walls, Cassandra L. Rigby, Wade J. VanderWright[1], Rima W. Jabado, Riley A. Pollom, John K. Carlson, Patricia Charvet, Ahmad Bin Ali, Fahmi, Jessica Cheok, Danielle H. Derrick, Katelyn B. Herman, Brittany Finucci, Tyler D. Eddy, Maria Lourdes D. Palomares, Christopher Avalos, Bineesh Kinattumkara, María-del-Pilar Blanco-Parra, Dharmadi, Mario Espinoza, Daniel Fernando, Alifa B. Haque, Paola A. Mejía-Falla, Andrés F. Navia, Juan Carlos Pérez-Jiménez, Jean Utzurrum, Ranny R. Yuneni, and Nicholas K. Dulvy[1]

Supplementary Notes

National Traits Predict Threat Level

Most of the national variation in extinction risk of coral reef sharks and rays is explained by abiotic factors related to population size, fishing pressure, and management capacity indices. The drivers of risk differed slightly between sharks and rays. The national extinction risk of sharks was more determined by the abiotic factors related to population size of sea surface temperature (19.8% [19.4–20.1%]), primary production (15.3% [14.9–15.6%]), and shelf area (15.2% [14.8–15.6%]); followed by fishing pressure indexed by coastal population (12.3% [12.0–12.6%]), marine protein consumption (9.1% [8.8–9.4%]), catch (3.6% [3.4–3.8%]), catch-per-unit-effort (CPUE) (0.9% [0.8–1.1%]), sightings-per-unit-effort (SPUE) (0.8% [0.7–0.9%]), and fishing effort (0.4% [0.3–0.5%]); and finally, management capacity indices of Gross Domestic Product (GDP) (11.3% [11.0–11.6%]), World Governance Index (WGI) (7.8% [7.5–8.1%]), and Human Development Index (HDI) (3.5% [3.3–3.7%]; Fig. 3). National extinction risk of rays was also determined by variable groupings in the same order of importance; however, management capacity indices were less than half the importance to rays as they were for sharks. Additionally, national extinction risk for rays was almost equally determined by ecological factors that affect carrying capacity and fishing pressure. The most important group of variables for rays were abiotic factors related to population size including sea surface temperature (32.4% [31.8–32.9%]), shelf area (10.1% [9.8–10.5%]), and primary production (4.1% [3.8–4.4%]); followed closely by fishing pressure indexed by CPUE (19.2% [18.7–19.6%]), catch (6.4% [6.1–6.8%]), marine protein consumption (5.6% [5.3–5.9%]), fishing effort (5.2% [4.9–5.6%]), SPUE (4.3% [4.1–4.6%]), and coastal population (2.8% [2.6–3.0%]). Finally, management capacity indices of HDI (4.4% [4.1–4.6%]), WGI (3.4% [3.1–3.6%]), and GDP (2.1% [1.9–2.4%]), were of little importance to rays (Fig. 3).

Where Are Reef Species Threatened?

The highest richness of coral reef sharks and rays combined occurs in the Indo-Pacific Coral Triangle with up to 52 species per 23,322 km² hexagonal grid cell (39% of all species; Supplementary Fig. 4a). This region is already well known for having the highest diversity of reef-building corals and coral reef teleosts [1,2]. The highest number of threatened species occurs in southern and southeast Asia with up to 42 species threatened per grid cell, reflecting the high levels of regional fisheries catch, effort, coastal population densities, and marine protein consumption (Supplementary Fig. 4b). However, the highest proportion of threatened reef sharks and rays occurs off the northeast coast of South America, the Andaman and Nicobar Islands, northeastern Taiwan, and western Africa (this area has few coral reefs but many transient, large-bodied, apex predator species that occur on coral reefs in other regions) (Supplementary Fig. 4c).

Similarly, the highest number of threatened species occurs in southern and Southeast Asia with up to 16 shark species and 26 ray species threatened within a single grid cell (Figs 4c,g). However, the region with the highest proportion of threatened sharks is the western Atlantic (Fig. 4c), possibly due to the comparatively low level of endemicity and diversity in the region. For rays, the proportion threatened is greatest throughout Asia and in southeast Africa with over 75% of species threatened in these areas (Fig. 4g). Reef rays have a higher proportion of their distribution where >90% of species within the map grid cell are threatened than sharks, suggesting rays have less spatial refuge from threats in reefs than sharks do (Figs 4d,h).

Supplementary Information References

1 Veron, J. E. N. *et al.* Delineating the Coral Triangle. *Galaxea, Journal of Coral Reef Studies* **11**, 91-100 (2009). https://doi.org:10.3755/galaxea.11.91

2 Asaad, I., Lundquist, C. J., Erdmann, M. V. & Costello, M. J. An interactive atlas for marine biodiversity conservation in the Coral Triangle. *Earth System Science Data* **11**, 163-174 (2019). https://doi.org:10.5194/essd-11-163-2019

3 MacNeil, M. A. *et al.* Global status and conservation potential of reef sharks. *Nature* **583**, 801-806 (2020). https://doi.org:10.1038/s41586-020-2519-y

Supplementary Figures

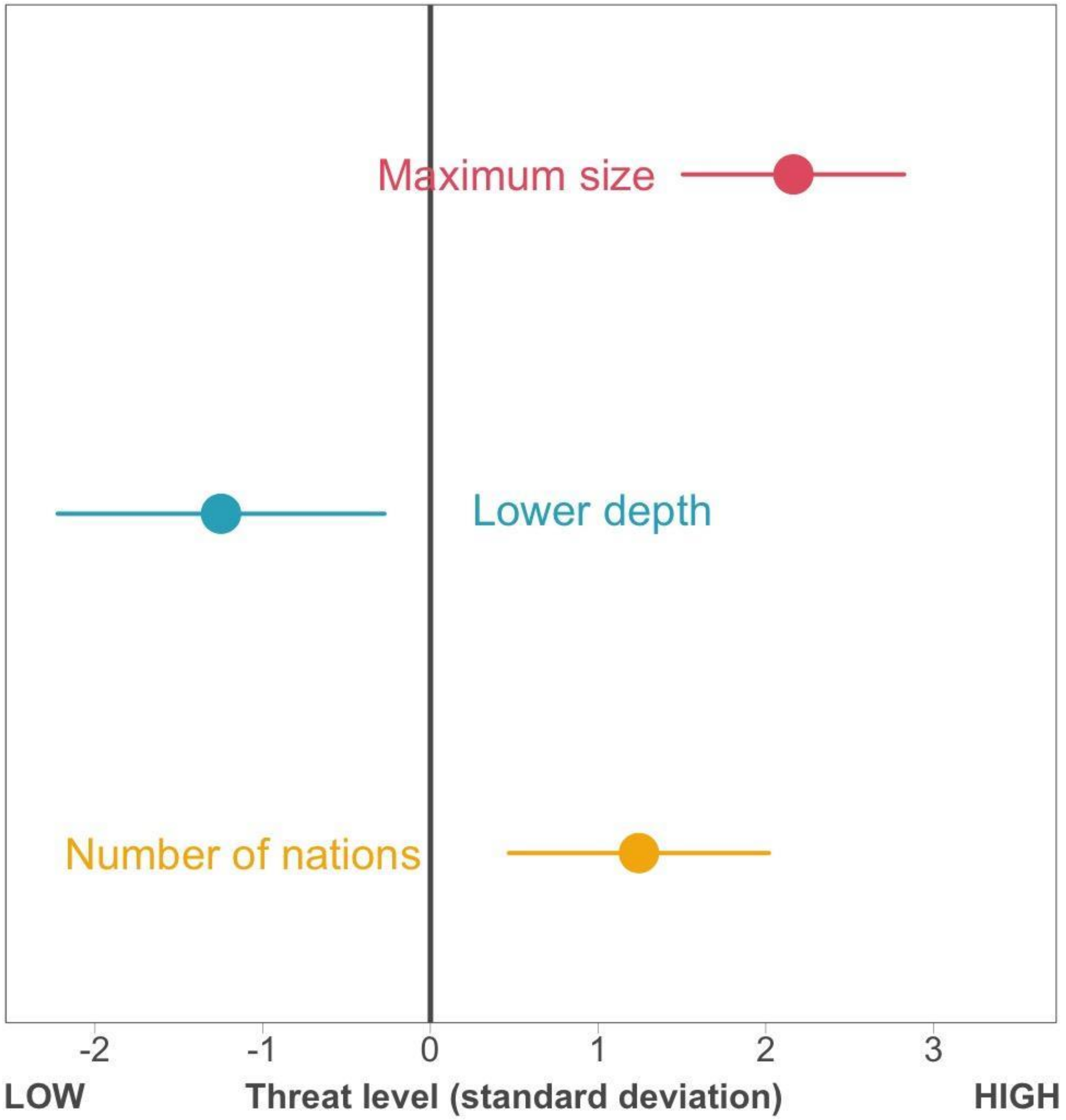

Supplementary Figure 1. Effects of body size, lower depth, and the number of nations a species occurs in, on the IUCN Red List status of sharks and rays. Circles represent the mean standardised effect sizes with bars representing 95% confidence intervals, n = 134 species.

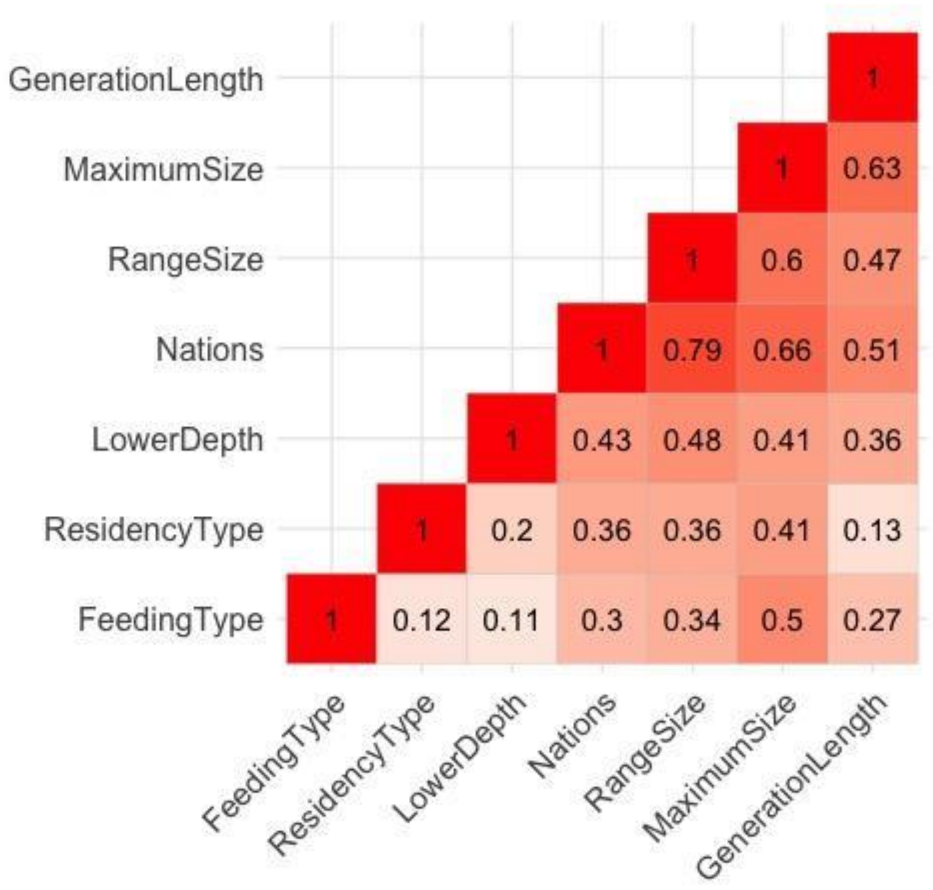

Supplementary Figure 2. Correlation matrix of all variables considered in the ordinal logistic regression models. While there is positive pairwise correlation between most pairs, those variables with correlation greater than 0.7 were not included in the same models. Empty boxes indicate pairwise relationships with negligible correlation. Other variables, though correlated, were included in additive models and tested for overall collinearity using variance inflation factors (VIF) <2.

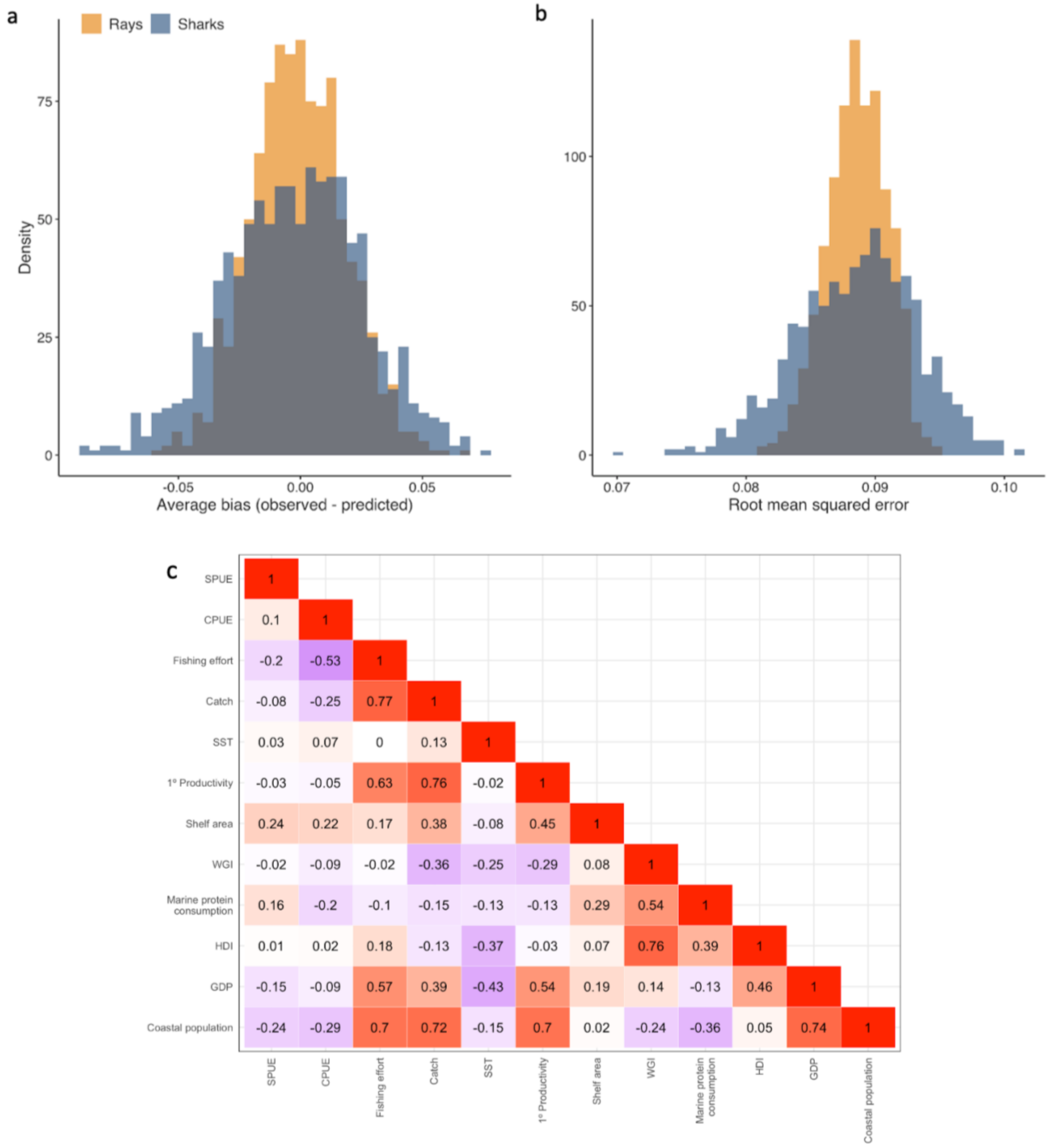

Supplementary Figure 3. Variation between each bootstrapped boosted regression tree iteration in the (a) average bias (calculated as the observed – predicted value from the test set) and (b) the root mean square error loss function for sharks and rays. (c) Correlations between national attributes.

Sharks and rays

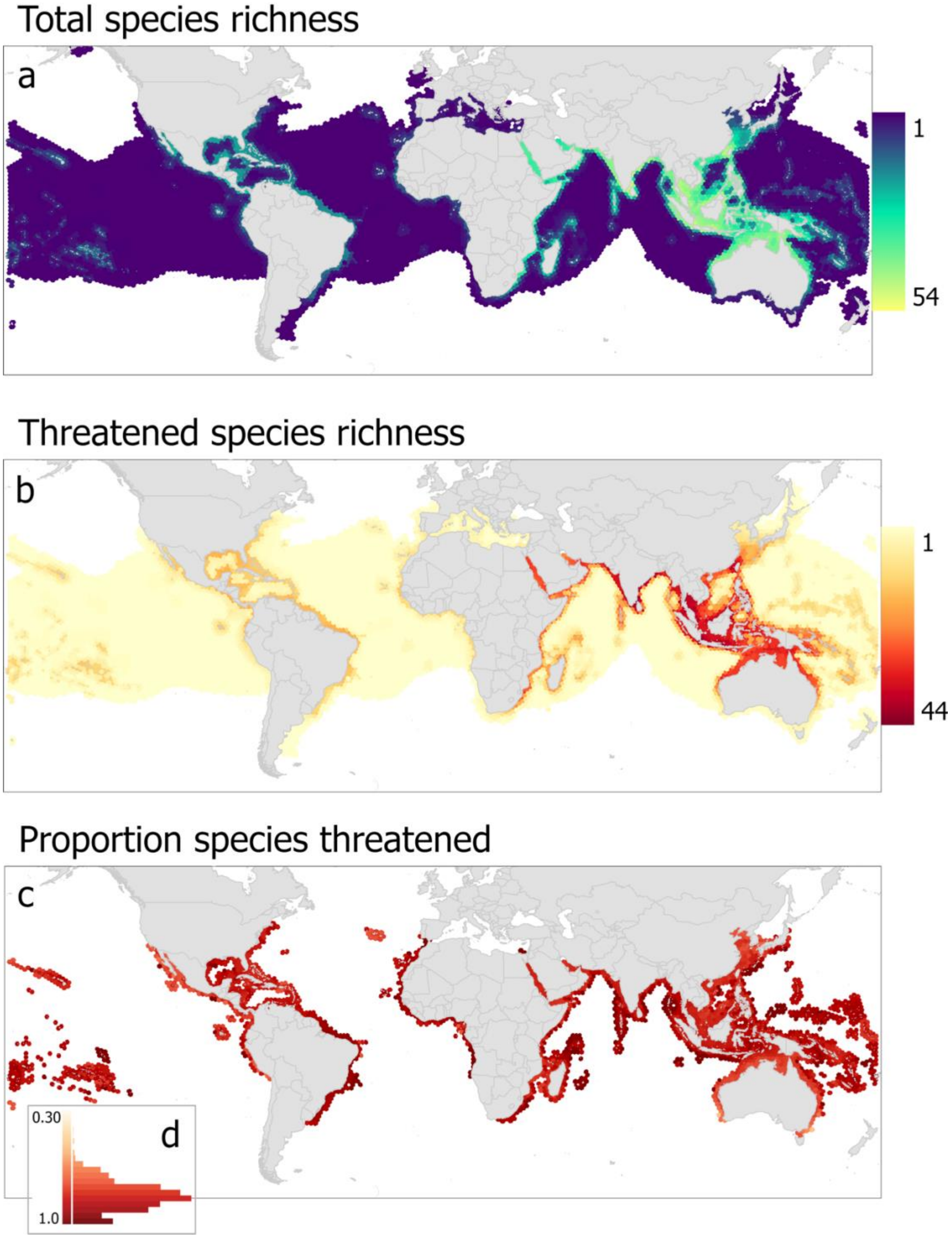

Supplementary Figure 4. Global combined shark and ray species richness, threat, and proportion threatened. (a) species richness, (b) number of threatened species, (c) threatened species as a proportion of total richness (for cells with >5 species), and (d) histograms represent the number of grid cells containing different percentages of threatened species.

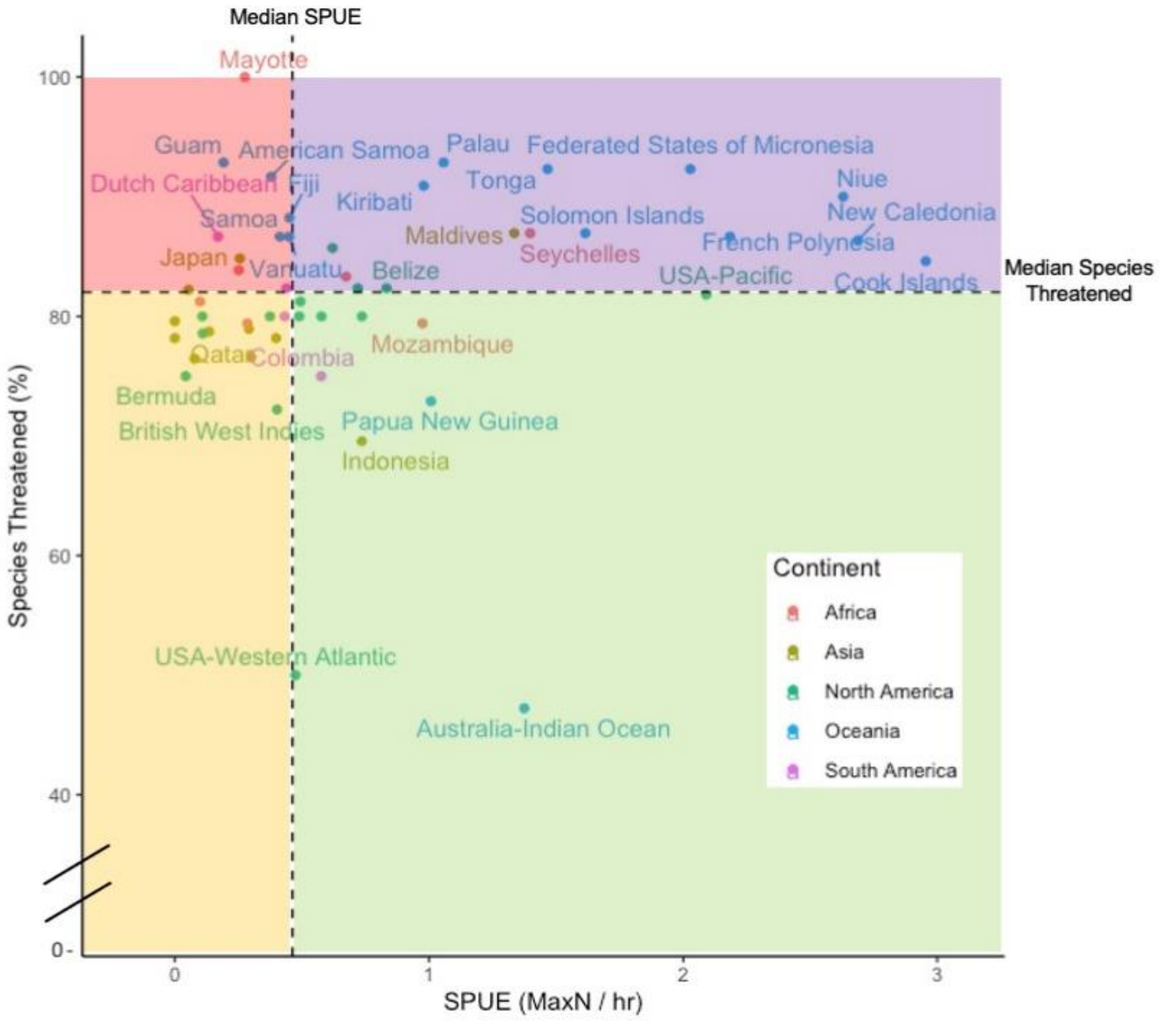

Supplementary Figure 5. Percent of coral reef sharks and rays threatened per nation compared to local abundance of sharks and rays over 371 coral reefs in 52 nations [3]. No significant relationship was found. Dotted lines indicate the median values of Sightings Per Unit Effort (SPUE) and Threat Percentage. SPUE was calculated by summing the total MaxN per nation and dividing this by the total hours of footage analysed. The proportion of species threatened was calculated by dividing the total number of Critically Endangered, Endangered, and Vulnerable species in a nation over the total number of coral reef sharks and rays in the nation. The purple quadrant represents nations with higher abundances of threatened species, acting as a refuge for these species. The red and yellow quadrants represent nations with low abundances of sharks and rays, and a high proportion of species threatened, and should be the focus of conservation capacity building. The green quadrant represents nations with fewer threatened species and higher abundances of sharks and rays.

Supplementary Tables

Supplementary Table 1. Percent of all 132 species threatened by Biological Resource Use (i.e., fishing) classified by intentionality (intentional vs. unintentional) and scale of the fishery (subsistence/small-scale vs. large-scale) separated by unique and pairwise, and combined threats. For unique threats, species are scored only when coded for the unique threat, or the column, or row pairwise combination. For the combined threats, species can be coded for up to all four combinations.

**Unique threat**	**Intentional (target catch)**	**Unintentional catch**	**Both intentional and unintentional**	**Scale total**
Subsistence/small scale	1.5% (2)	3.0% (4)	9.1% (12)	13.6% (18)
Large-scale	0.0% (0)	4.5% (6)	0.8% (1)	5.3% (7)
Both subsistence and large-scale	0.0% (0)	24.2% (32)		
Intentionality total	1.5% (2)	31.8% (42)		
**Combined threats**	**Intentional (target catch)**	**Unintentional catch**		**Scale total**
Subsistence/small scale	64.4% (85)	91.7% (121)		94.7% (125)
Large-scale	40.9% (54)	85.6% (113)		86.4% (114)
Intentionality total	68.2% (90)	98.5% (130)		

Supplementary Table 2. Observed number and percent of shark and ray species in IUCN Red List categories separated for lineages - rays (Subclass Batoidea), sharks (Subclass Selachimorpha), and combined, for residency type - transient, partial resident, resident, and for trophic level - apex predator, mesopredator, filter feeder, large invertivores, and small invertivores.

	Species Number (% of 134)	Number threatened (% of 79)	Number threatened (% of group)	CR (%)	EN (%)	VU (%)	NT (%)	LC (%)	DD (%)
**Taxon**									
Rays	70 (52.2)	45/79 (57.0)	45/70 (64.3)	9 (12.8)	14 (20.0)	22 (31.4)	6 (8.6)	13 (18.6)	6 (8.6)
Sharks	64 (47.8)	34/79 (43.0)	34/64 (53.1)	5 (7.8)	10 (15.6)	19 (29.7)	12 (18.8)	15 (23.4)	3 (4.7)
Total	134		79/134 (59.0)	14 (10.5)	24 (17.9)	41 (30.6)	18 (13.4)	28 (20.9)	9 (6.7)
**Residency**									
Transient	25 (18.7)	19/79 (24.1)	19/25 (76.0)	2 (8.0)	11 (44.0)	6 (24.0)	3 (12.0)	1 (4.0)	2 (8.0)
Partial Resident	54 (40.3)	36/79 (45.6)	36/54 (66.7)	11 (20.4)	6 (11.1)	19 (35.2)	6 (11.1)	10 (18.2)	2 (3.7)
Resident	55 (41.0)	24/79 (30.4)	24/55 (43.6)	1 (1.8)	7 (12.7)	16 (29.1)	9 (16.4)	17 (30.9)	5 (9.1)
**Trophic Level**									
Apex	6 (4.5)	5/79 (6.3)	5/6 (83.3)	2 (33.3)	1 (16.7)	2 (33.3)	1 (16.7)	0 (0.0)	0 (0.0)

Mesopredator	41 (30.6)	27/79 (34.2)	27/41 (65.9)	10 (24.4)	9 (22.0)	8 (19.5)	4 (9.8)	9 (22.0)	1 (2.4)
Filter Feeder	7 (5.2)	7/79 (8.9)	7/7 (100.0)	0 (0.0)	6 (85.7)	1 (14.3)	0 (0.0)	0 (0.0)	0 (0.0)
Large Invertivores	19 (14.2)	18/79 (22.8)	18/19 (94.7)	0 (0.0)	5 (26.3)	13 (68.4)	1 (5.3)	0 (0.0)	0 (0.0)
Small Invertivores	61 (45.5)	22/79 (27.8)	22/61 (36.1)	2 (3.3)	3 (4.9)	17 (27.9)	12 (19.7)	19 (31.1)	8 (13.1)

Supplementary Table 3. All models trialled in order of weighting, including their difference in AIC from the top model (ΔAIC), AIC weight, and the largest variance inflation factor (VIF) value. Only models with VIF values <2 were considered, models not considered are in grey.

Model	ΔAIC	Weight	VIF
Maximum Linear Dimension + Lower Depth + Nations	0	0.78	1.86
Maximum Linear Dimension + Lower Depth	4.42	0.09	1.20
Maximum Linear Dimension + Lower Depth + Residency	4.98	0.06	1.40
Maximum Linear Dimension + Ordinal Residency	8.08	0.01	1.21
Maximum Linear Dimension + Nations	8.43	0.01	1.76
Maximum Linear Dimension + Generation Length + Nations	9.40	0.01	2.22
Maximum Linear Dimension + Residency + Range	9.46	0.01	1.30
Maximum Linear Dimension + Range	9.56	0.01	1.57
Maximum Linear Dimension + Residency	9.74	0.01	1.10
Maximum Linear Dimension + Nations + Ordinal Residency + Ordinal Trophic Group	10.25	0	2.32
Maximum Linear Dimension + Generation Length	10.32	0	1.65
Maximum Linear Dimension	10.54	0	-
Maximum Linear Dimension + Ordinal Trophic Group	10.94	0	1.34
Maximum Linear Dimension + Generation Length + Range	11.08	0	2.06
Maximum Linear Dimension + Generation Length + Residency	11.24	0	1.44
Maximum Linear Dimension + Trophic Group	16.53	0	1.67
Maximum Linear Dimension + Trophic Group + Generation Length	17.71	0	1.84
Nations	21.36	0	-

Nations + Ordinal Trophic Group	21.73	0	1.10
Generation Length + Nations	22.71	0	1.34
Trophic Group + Nations	23.25	0	1.32
Trophic Group	27.79	0	-
Residency + Trophic Group	28.86	0	1.13
Nations + Ordinal Residency	29.07	0	1.15
Trophic Group + Range	29.21	0	1.31
Residency + Generation Length	30.81	0	1.04
Residency	36.98	0	-
Generation Length + Range	38.69	0	1.28
Residency + Lower Depth	38.98	0	1.03
Generation Length	39.41	0	-
Generation Length + Lower Depth	41.16	0	1.15
Ocean Basin	43.50	0	-
Shark or Ray	46.93	0	-
Null	47.54	0	-
Threat of Climate Change	47.64	0	-
Lower Depth	49.04	0	-

Supplementary Table 4. Species with 'coral reef' habitat listed in their IUCN Red List assessment but omitted from this analysis and the reason for their exclusion.

**Species**	**Reason Not To Include**
Aulohalaelurus labiosus	Occurs on temperate rocky reefs, distribution does not include coral reefs
Carcharhinus hemiodon	Occurs in estuaries and soft bottom coastal habitats
Carcharhinus longimanus	Occurs in open ocean pelagic habitats
Fontitrygon margaritella	Range is in western Africa, where there are almost no coral reefs
Galeus mincaronei	Occurs only on deep reefs >130 m
Orectolobus floridus	Occurs on temperate rocky reefs, distribution does not include coral reefs
Rhynchobatus luebberti	Range is in western Africa, where there are almost no coral reefs
Schroederichthys saurisqualus	Occurs on deep-water reefs off the coast of Brazil
Scyliorhinus haeckelii	Occurs on deep-water reefs
Scyliorhinus stellaris	Range is in Europe and western Africa, where there are almost no coral reefs
Sutorectus tentaculatus	Occurs on temperate rocky reefs, distribution does not include coral reefs
Triakis maculata	Occurs on temperate rocky reefs, distribution does not include coral reefs

Supplementary Table 5. Species included as 'coral reef species' despite their IUCN Red List assessments not including this in their list of habitats.

**Scientific Name**	**Common Name**
Aetobatus laticeps	pacific eagle ray
Chiloscyllium caeruleopunctatum	bluespotted bambooshark
Heterodontus japonicus	Japanese bullhead shark
Hypanus longus	longtail stingray
Maculabatis ambigua	Baraka's whipray
Narcine entemedor	Cortez numbfish
Neotrygon annotata	plain maskray
Neotrygon kuhlii	Kuhl's maskray
Neotrygon picta	speckled maskray
Pristis pectinata	smalltooth sawfish
Rhincodon typus	whale shark
Rhynchobatus laevis	smoothnose wedgefish
Sphyrna lewini	scalloped hammerhead
Sphryna mokarran	great hammerhead
Styracura pacifica	Pacific chupare
Triakis scyllium	banded houndshark
Urobatis halleri	round stingray
Urogymnus granulatus	mangrove whipray
Urotrygon chilensis	Chilean round ray
Zapteryx xyster	southern banded guitarfish

Supplementary Table 6. Species that do not have previous assessments online due to revised taxonomic concept since their first assessment, but that we have not classified as previously Not Evaluated.

**Species**	**Previous Taxonomic Concept**	**Revised Taxonomic Concept**
Aetobatus narinari	NT (2006)	EN (2020)
Aetomylaeus nichofii	VU (2003)	VU (2016)
Bathytoshia lata	LC (2007)	VU (2021)
Chiloscyllium plagiosum	NT (2006)	NT (2020)
Ginglymostoma cirratum	DD (2006)	VU (2019)
Himantura uarnak	VU (2015)	EN (2021)
Hypanus americanus	DD (2006)	NT (2019)
Neotrygon kuhlii	DD (2015)	DD (2018)
Taeniura lymma	NT (2005)	LC (2020)
