# Supplementary material for: Half a century of rising extinction risk of coral reef sharks and rays
Source: Nat Commun. 2023 Jan 17;14:15. doi: 10.1038/s41467-022-35091-x (PMC9845228; doi:10.1038/s41467-022-35091-x)
Supplement: Supplementary file 3 — Description of Additional Supplementary Files [file 41467_2022_35091_MOESM3_ESM.pdf]

Description of Additional Supplementary Files

File Name: Supplementary Data 1.

Description: Data sources available on the IUCN Red List of Threatened Species, related to methods.

File Name: Supplementary Data 2.

Description: Species residency, trophic level, and generation lengths and their derivation for all species included, related to methods and their retrospective Red List status' for 2005, 1980, and 1970.

File Name: Supplementary Data 3.

Description: List of 153 Assessors, 148 Contributors, 37 Reviewers, and 18 Facilitators in alphabetical order of first name.

File Name: Supplementary Data 4.

Description: Covariate values used for the national trait boosted regression tree analysis along with the source of these values.
