# Supplementary material for: Half a century of rising extinction risk of coral reef sharks and rays
Source: Nat Commun. 2023 Jan 17;14:15. doi: 10.1038/s41467-022-35091-x (PMC9845228; doi:10.1038/s41467-022-35091-x)
Supplement: Supplementary file 8 — Reporting Summary [file 41467_2022_35091_MOESM8_ESM.pdf]

nature portfolio

Corresponding author(s): C. Samantha Sherman
Colin A. Simpfendorfer

Last updated by author(s): November 5, 2022

Reporting Summary

Nature Portfolio wishes to improve the reproducibility of the work that we publish. This form provides structure for consistency and transparency in reporting. For further information on Nature Portfolio policies, see our Editorial Policies and the Editorial Policy Checklist.

Statistics

For all statistical analyses, confirm that the following items are present in the figure legend, table legend, main text, or Methods section.

n/a	Confirmed	
☐	☒	The exact sample size (*n*) for each experimental group/condition, given as a discrete number and unit of measurement
☒	☐	A statement on whether measurements were taken from distinct samples or whether the same sample was measured repeatedly
☒	☐	The statistical test(s) used AND whether they are one- or two-sided<br>*Only common tests should be described solely by name; describe more complex techniques in the Methods section.*
☐	☒	A description of all covariates tested
☐	☒	A description of any assumptions or corrections, such as tests of normality and adjustment for multiple comparisons
☐	☒	A full description of the statistical parameters including central tendency (e.g. means) or other basic estimates (e.g. regression coefficient) AND variation (e.g. standard deviation) or associated estimates of uncertainty (e.g. confidence intervals)
☒	☐	For null hypothesis testing, the test statistic (e.g. *F*, *t*, *r*) with confidence intervals, effect sizes, degrees of freedom and *P* value noted<br>*Give P values as exact values whenever suitable.*
☒	☐	For Bayesian analysis, information on the choice of priors and Markov chain Monte Carlo settings
☒	☐	For hierarchical and complex designs, identification of the appropriate level for tests and full reporting of outcomes
☐	☒	Estimates of effect sizes (e.g. Cohen's *d*, Pearson's *r*), indicating how they were calculated

Our web collection on statistics for biologists contains articles on many of the points above.

Software and code

Policy information about availability of computer code

Data collection	No software was used in data collection.
Data analysis	All analyses were performed using R Statistical Software v.4.1.0 using the following packages: ordinal v.2019.12.10, PerformanceAnalytics v.2.0.4, coefplot v.1.2.8, XGBoost v.1.4.1.1, and betareg v.3.1-4.

For manuscripts utilizing custom algorithms or software that are central to the research but not yet described in published literature, software must be made available to editors and reviewers. We strongly encourage code deposition in a community repository (e.g. GitHub). See the Nature Portfolio guidelines for submitting code & software for further information.

Data

Policy information about availability of data

All manuscripts must include a data availability statement. This statement should provide the following information, where applicable:

- Accession codes, unique identifiers, or web links for publicly available datasets
- A description of any restrictions on data availability
- For clinical datasets or third party data, please ensure that the statement adheres to our policy

IUCN Red List of Threatened Species assessments are publicly available with links to specific assessments in Supplementary Table 1.

Field-specific reporting

Please select the one below that is the best fit for your research. If you are not sure, read the appropriate sections before making your selection.

☐ Life sciences ☐ Behavioural & social sciences ☒ Ecological, evolutionary & environmental sciences

For a reference copy of the document with all sections, see nature.com/documents/nr-reporting-summary-flat.pdf

Ecological, evolutionary & environmental sciences study design

All studies must disclose on these points even when the disclosure is negative.

Study description	We gathered data from the IUCN Red List of Threatened Species for coral reef associated shark and ray species. We then analysed their extinction risk based on species traits, national traits, and threat categories.
Research sample	Species were selected based on their occurrence on coral reefs at some point during their lives and their inclusion on the IUCN Red List of Threatened Species. All data was sourced from the IUCN Red List website (www.redlist.org)
Sampling strategy	The IUCN Red List website was searched using the "advanced search" function (www.iucnredlist.org). The search included all species in Class "Chondrichthyes" with the habitat "9.8 - Marine Neritic - Coral Reef." This query selected all known shark and ray species that occur on coral reefs. This meant that we had comprehensive information on this taxa from coral reefs. The Red List is the most globally comprehensive and complete repository of information on the status and threats of species.
Data collection	Data were extracted from relevant online data repositories or scientific literature by Dr. C. Samantha Sherman. Species-specific attributes (maximum size, generation length, geographic range, number of nations they occur in, lower depth limit) were gathered from their respective IUCN Red List Assessments where possible. Generation lengths were not always available and were estimated using proxy data from related species when absent. The species traits of residency pattern and trophic level were estimated through expert opinion.
Timing and spatial scale	The earliest IUCN Red List assessment used in this study was from 2013, with the most recent assessments being published in 2021. A final search for coral reef sharks and rays was completed on April 17, 2021. The Red List Index was based on hind-cast extinction risk estimates back to 1970. All data were based on the global extent of each species.
Data exclusions	Data-deficient species were excluded from Red List Index analyses. Twelve species from the IUCN Red List search were not included for various reasons (Extended Data Table 3).
Reproducibility	This is not applicable in this study as the data and analysis methods are all publicly available. There was no experimentation in the study.
Randomization	This was not relevant for our study, no randomization was needed. All covariates were linked either to a species or nation, therefore, randomization would have been inappropriate.
Blinding	Blinding was not appropriate for this work - this study did not involve experiments, and the analyses did not require blinding.

Did the study involve field work? ☐ Yes ☒ No

Reporting for specific materials, systems and methods

We require information from authors about some types of materials, experimental systems and methods used in many studies. Here, indicate whether each material, system or method listed is relevant to your study. If you are not sure if a list item applies to your research, read the appropriate section before selecting a response.

Materials & experimental systems

n/a	Involved in the study	
☒	☐	Antibodies
☒	☐	Eukaryotic cell lines
☒	☐	Palaeontology and archaeology
☒	☐	Animals and other organisms
☒	☐	Human research participants
☒	☐	Clinical data
☒	☐	Dual use research of concern

Methods

n/a	Involved in the study	
☒	☐	ChIP-seq
☒	☐	Flow cytometry
☒	☐	MRI-based neuroimaging
